# Chlorido[2-({[2-(diphenyl­phosphan­yl)­benzyl­idene]amino}­meth­yl)thio­phene-κ^2^ *N*,*P*]methyl­palladium(II)

**DOI:** 10.1107/S1600536812007295

**Published:** 2012-02-29

**Authors:** William M. Motswainyana, Martin O. Onani, Roger A. Lalancette

**Affiliations:** a Chemistry Department, University of the Western Cape, Modderdam Road, Private Bag X17, Bellville 7535, South Africa; b Carl A. Olson Memorial Laboratories, Department of Chemistry, Rutgers University, Newark, NJ 07102, USA

## Abstract

In the title compound, [Pd(CH_3_)Cl(C_24_H_20_NPS)], the Pd^II^ ion is coordinated in a distorted square-planar environment which includes the P and N atoms of the bis-chelating ligand. The thio­phene ring is rotationally ordered, unlike in the majority of crystal structures containing this group.

## Related literature

For the synthesis of imino-phosphine ligands and their transition metal-based complexes, see: Nobre & Monteiro (2009 ▶); Pelagatti *et al.* (2005 ▶); Reddy *et al.* (2001 ▶); Espinet & Soulantica (1999 ▶). For related structures, see: Onani *et al.* (2010 ▶); Vaughan *et al.* (2011 ▶).
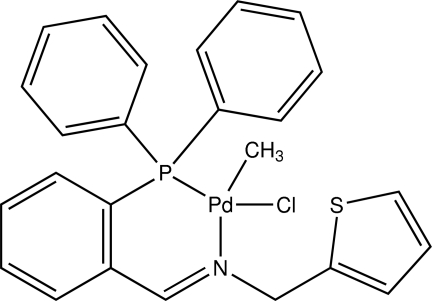


## Experimental

### 

#### Crystal data

[Pd(CH_3_)Cl(C_24_H_20_NPS)]
*M* *_r_* = 542.32
Monoclinic, 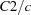

*a* = 24.6534 (4) Å
*b* = 10.0118 (1) Å
*c* = 18.4507 (3) Å
β = 98.027 (1)°
*V* = 4509.47 (11) Å^3^
*Z* = 8
Cu *K*α radiation
μ = 9.35 mm^−1^
*T* = 100 K
0.26 × 0.15 × 0.08 mm

#### Data collection

Bruker SMART CCD APEXII diffractometer
Absorption correction: numerical (*SADABS*; Sheldrick, 2008*a* ▶) *T*_min_ = 0.195, *T*_max_ = 0.522
22554 measured reflections
4048 independent reflections
3919 reflections with *I* > 2σ(*I*)
*R*_int_ = 0.028

#### Refinement

*R*[*F* ^2^ > 2σ(*F* ^2^)] = 0.024
*wR*(*F* ^2^) = 0.062
*S* = 1.11
4048 reflections
272 parameters
H-atom parameters constrained
Δρ_max_ = 0.50 e Å^−3^
Δρ_min_ = −0.44 e Å^−3^

### 

Data collection: *APEX2* (Bruker, 2006 ▶); cell refinement: *APEX2*; data reduction: *SAINT* (Bruker, 2005 ▶); program(s) used to solve structure: *SHELXTL* (Sheldrick, 2008*b* ▶); program(s) used to refine structure: *SHELXTL*; molecular graphics: *SHELXTL*; software used to prepare material for publication: *SHELXTL*.

## Supplementary Material

Crystal structure: contains datablock(s) I, New_Global_Publ_Block, global. DOI: 10.1107/S1600536812007295/lh5417sup1.cif

Structure factors: contains datablock(s) I. DOI: 10.1107/S1600536812007295/lh5417Isup2.hkl

Additional supplementary materials:  crystallographic information; 3D view; checkCIF report

## Figures and Tables

**Fig. 1.**
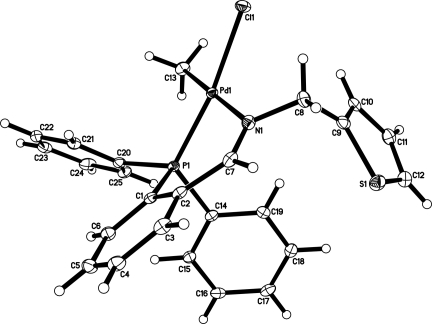
The molecular structure of (I). Displacement ellipsoids are drawn at the 40% probability level for non-H atoms.

## supplementary crystallographic information

### Comment

Iminophosphine complexes are easily prepared by Schiff base condensation reactions. These complexes are widely used for C—C coupling reactions in organic synthesis (Reddy *et al.*, 2001; Pelagatti *et al.*, 2005). They are better utilized for aromatic carbon coupling type of reactions due to their reaction mode. Basically, the iminophosphine ligand possesses hard nitrogen and soft phosphorus donor atoms that impart the unique property of hemilability. The N—P combination brings about asymmetry in the Pd orbitals thereby affecting the reactivity of a complex. An investigation of the catalytic mechanism revealed that the hemilabile ligand present in the complex is accountable for the catalytic cycle because it allows the inflection of the steric properties around Pd, which determines the activity and selectivity parameters of the complexes containing these ligands (Espinet & Soulantica, 1999; Onani *et al.*, 2010; Vaughan *et al.*, 2011). The title compound is a bidentate and bulky complex that should be highly active for C—C coupling studies. Some of these types of complexes have been described as therapeutic agents (Nobre & Monteiro, 2009).

The molecular structure of the title compound (I) is shown in Fig 1. The Pd^II^ ion is coordinated in a bidentate mode to the P and N atoms of the iminophosphine ligand. The coordination is completed by chloride and methyl ligands. The bond angles; Cl1—Pd1—P1 [171.99 (2)°], Cl1—Pd1—N1 [93.96 (6)°], Cl1—Pd1—C13 [86.45 (8)°] and P1—Pd1—N1 [90.23 (6)°] describe a distorted square planar coordination geometry around the metal center.

### Experimental

Pd(COD)ClMe (0.0545 g, 0.206 mmol) was added to a Schlenk tube charged with 15 ml of CH_2_Cl_2_/Et_2_O solution (1:2). A ligand of 2-(diphenylphosphino)benzyl-2-thiophenemethylimine (0.0762 g, 0.206 mmol) was dissolved separately in 2 ml dichloromethane and the resultant solution was added dropwise to a Schlenk tube containing the metal precursor. The reaction mixture was stirred at room temperature for 8 hrs, resulting in the formation of a white precipitate. This precipitate was filtered to obtain a white solid, which formed shiny white crystals suitable for X-ray analysis when recrystallized from a mixture of a minimum amount of CH_2_Cl_2_ and an excess of C_6_H_14_.

### Refinement

All H atoms were found in electron density difference maps. Subsequently, the methyl H atoms were placed in ideally staggered positions with C—H distances of 0.98 Å and *U*_iso_(H) = 1.5*U*_eq_(C). The methylene, methine, phenyl and thiophenyl H atoms were placed in geometrically idealized positions and constrained to ride on their parent C atoms with C—H distances of 0.99, 1.00, 0.95, and 0.95 Å respectively, and *U*_iso_(H) = 1.2*U*_eq_(C). The low fraction of data collected may affect the precision of the structure.

### Crystal data

**Table:**

[Pd(CH_3_)Cl(C_24_H_20_NPS)]	*F*(000) = 2192
*M**_r_* = 542.32	*D*_x_ = 1.598 Mg m^−^^3^
Monoclinic, *C*2/*c*	Cu *K*α radiation, λ = 1.54178 Å
Hall symbol: -C 2yc	Cell parameters from 9937 reflections
*a* = 24.6534 (4) Å	θ = 3.6–72.0°
*b* = 10.0118 (1) Å	µ = 9.35 mm^−^^1^
*c* = 18.4507 (3) Å	*T* = 100 K
β = 98.027 (1)°	Plate, yellow
*V* = 4509.47 (11) Å^3^	0.26 × 0.15 × 0.08 mm
*Z* = 8	

### Data collection

**Table:**

Bruker SMART CCD APEXII diffractometer	4048 independent reflections
Radiation source: fine-focus sealed tube	3919 reflections with *I* > 2σ(*I*)
Graphite monochromator	*R*_int_ = 0.028
φ and ω scans	θ_max_ = 71.9°, θ_min_ = 3.6°
Absorption correction: numerical (*SADABS*; Sheldrick, 2008*a*)	*h* = −29→28
*T*_min_ = 0.195, *T*_max_ = 0.522	*k* = −11→11
22554 measured reflections	*l* = −21→22

### Refinement

**Table:**

Refinement on *F*^2^	Primary atom site location: structure-invariant direct methods
Least-squares matrix: full	Secondary atom site location: difference Fourier map
*R*[*F*^2^ > 2σ(*F*^2^)] = 0.024	Hydrogen site location: inferred from neighbouring sites
*wR*(*F*^2^) = 0.062	H-atom parameters constrained
*S* = 1.11	*w* = 1/[σ^2^(*F*_o_^2^) + (0.0291*P*)^2^ + 12.1978*P*] where *P* = (*F*_o_^2^ + 2*F*_c_^2^)/3
4048 reflections	(Δ/σ)_max_ < 0.001
272 parameters	Δρ_max_ = 0.50 e Å^−^^3^
0 restraints	Δρ_min_ = −0.44 e Å^−^^3^

### Special details

**Table:**

Experimental. crystal mounted on a Cryoloop using Paratone-N
Geometry. All e.s.d.'s (except the e.s.d. in the dihedral angle between two l.s. planes) are estimated using the full covariance matrix. The cell e.s.d.'s are taken into account individually in the estimation of e.s.d.'s in distances, angles and torsion angles; correlations between e.s.d.'s in cell parameters are only used when they are defined by crystal symmetry. An approximate (isotropic) treatment of cell e.s.d.'s is used for estimating e.s.d.'s involving l.s. planes.
Refinement. Refinement of *F*^2^ against ALL reflections. The weighted *R*-factor *wR* and goodness of fit *S* are based on *F*^2^, conventional *R*-factors *R* are based on *F*, with *F* set to zero for negative *F*^2^. The threshold expression of *F*^2^ > σ(*F*^2^) is used only for calculating *R*-factors(gt) *etc*. and is not relevant to the choice of reflections for refinement. *R*-factors based on *F*^2^ are statistically about twice as large as those based on *F*, and *R*- factors based on ALL data will be even larger.

### Fractional atomic coordinates and isotropic or equivalent isotropic displacement parameters (Å^2^)

**Table:**

	*x*	*y*	*z*	*U*_iso_*/*U*_eq_	
Pd1	0.111634 (7)	0.271512 (17)	0.253204 (8)	0.01132 (7)	
Cl1	0.10246 (2)	0.09406 (6)	0.33614 (3)	0.02005 (13)	
S1	0.08000 (3)	−0.00649 (6)	0.02911 (3)	0.02051 (14)	
P1	0.11772 (2)	0.45419 (6)	0.18890 (3)	0.01083 (12)	
N1	0.04735 (8)	0.1992 (2)	0.17213 (11)	0.0150 (4)	
C1	0.05000 (9)	0.5072 (2)	0.14458 (12)	0.0125 (5)	
C2	0.00859 (9)	0.4126 (2)	0.12111 (12)	0.0140 (5)	
C3	−0.04027 (10)	0.4576 (3)	0.08017 (13)	0.0178 (5)	
H3	−0.0682	0.3948	0.0637	0.021*	
C4	−0.04872 (10)	0.5906 (3)	0.06324 (13)	0.0194 (5)	
H4	−0.0823	0.6188	0.0359	0.023*	
C5	−0.00817 (10)	0.6833 (3)	0.08607 (14)	0.0192 (5)	
H5	−0.0138	0.7752	0.0744	0.023*	
C6	0.04080 (10)	0.6410 (3)	0.12609 (13)	0.0161 (5)	
H6	0.0686	0.7048	0.1412	0.019*	
C7	0.01250 (10)	0.2671 (2)	0.12986 (13)	0.0153 (5)	
H7	−0.0146	0.2167	0.0999	0.018*	
C8	0.04345 (10)	0.0513 (2)	0.16373 (14)	0.0180 (5)	
H8A	0.0075	0.0272	0.1361	0.022*	
H8B	0.0464	0.0089	0.2126	0.022*	
C9	0.08836 (10)	0.0011 (2)	0.12399 (13)	0.0162 (5)	
C10	0.14046 (10)	−0.0368 (2)	0.15316 (14)	0.0178 (5)	
H10	0.1533	−0.0392	0.2041	0.021*	
C11	0.17293 (10)	−0.0723 (3)	0.09775 (14)	0.0197 (5)	
H11	0.2099	−0.1013	0.1083	0.024*	
C12	0.14600 (11)	−0.0609 (3)	0.02862 (15)	0.0217 (5)	
H12	0.1616	−0.0804	−0.0144	0.026*	
C13	0.17473 (10)	0.3382 (3)	0.32856 (13)	0.0194 (5)	
H13A	0.1928	0.2619	0.3550	0.029*	
H13B	0.1603	0.3981	0.3633	0.029*	
H13C	0.2012	0.3865	0.3034	0.029*	
C14	0.15438 (9)	0.4419 (2)	0.10996 (12)	0.0127 (5)	
C15	0.16901 (10)	0.5568 (3)	0.07414 (13)	0.0160 (5)	
H15	0.1619	0.6427	0.0926	0.019*	
C16	0.19398 (10)	0.5452 (3)	0.01147 (13)	0.0181 (5)	
H16	0.2048	0.6232	−0.0122	0.022*	
C17	0.20320 (10)	0.4202 (3)	−0.01671 (13)	0.0179 (5)	
H17	0.2198	0.4127	−0.0600	0.021*	
C18	0.18818 (9)	0.3060 (3)	0.01822 (13)	0.0156 (5)	
H18	0.1942	0.2205	−0.0015	0.019*	
C19	0.16432 (9)	0.3164 (2)	0.08196 (13)	0.0133 (5)	
H19	0.1548	0.2380	0.1064	0.016*	
C20	0.14646 (10)	0.5991 (2)	0.23982 (12)	0.0138 (5)	
C21	0.11485 (10)	0.6668 (2)	0.28528 (13)	0.0156 (5)	
H21	0.0776	0.6430	0.2853	0.019*	
C22	0.13771 (11)	0.7685 (2)	0.33021 (14)	0.0190 (5)	
H22	0.1159	0.8149	0.3606	0.023*	
C23	0.19234 (11)	0.8030 (3)	0.33115 (13)	0.0203 (5)	
H23	0.2078	0.8738	0.3616	0.024*	
C24	0.22424 (11)	0.7342 (3)	0.28783 (14)	0.0202 (5)	
H24	0.2618	0.7567	0.2893	0.024*	
C25	0.20175 (10)	0.6324 (3)	0.24205 (13)	0.0169 (5)	
H25	0.2239	0.5856	0.2123	0.020*	

### Atomic displacement parameters (Å^2^)

**Table:**

	*U*^11^	*U*^22^	*U*^33^	*U*^12^	*U*^13^	*U*^23^
Pd1	0.01188 (10)	0.01295 (11)	0.00892 (10)	0.00129 (6)	0.00075 (7)	0.00036 (6)
Cl1	0.0251 (3)	0.0184 (3)	0.0173 (3)	0.0039 (2)	0.0054 (2)	0.0064 (2)
S1	0.0201 (3)	0.0235 (3)	0.0165 (3)	0.0007 (2)	−0.0027 (2)	−0.0013 (2)
P1	0.0099 (3)	0.0129 (3)	0.0091 (3)	−0.0002 (2)	−0.0006 (2)	−0.0002 (2)
N1	0.0142 (10)	0.0149 (10)	0.0162 (10)	−0.0028 (8)	0.0028 (8)	−0.0019 (8)
C1	0.0117 (11)	0.0174 (12)	0.0083 (10)	0.0009 (9)	0.0007 (9)	−0.0002 (9)
C2	0.0117 (11)	0.0205 (13)	0.0101 (11)	−0.0001 (9)	0.0027 (9)	−0.0012 (9)
C3	0.0115 (11)	0.0260 (14)	0.0156 (12)	−0.0014 (10)	0.0012 (10)	−0.0017 (10)
C4	0.0133 (12)	0.0299 (14)	0.0143 (12)	0.0058 (10)	−0.0010 (10)	0.0014 (10)
C5	0.0204 (13)	0.0197 (13)	0.0178 (12)	0.0043 (10)	0.0032 (10)	0.0038 (10)
C6	0.0146 (12)	0.0186 (13)	0.0146 (11)	−0.0005 (9)	0.0005 (10)	−0.0001 (10)
C7	0.0113 (12)	0.0205 (13)	0.0145 (12)	−0.0027 (9)	0.0032 (10)	−0.0036 (9)
C8	0.0187 (12)	0.0134 (12)	0.0218 (13)	−0.0023 (10)	0.0027 (10)	−0.0017 (10)
C9	0.0194 (12)	0.0124 (12)	0.0158 (12)	−0.0040 (9)	−0.0013 (10)	0.0011 (9)
C10	0.0236 (13)	0.0099 (12)	0.0205 (12)	0.0000 (10)	0.0048 (11)	0.0024 (10)
C11	0.0177 (12)	0.0160 (13)	0.0248 (13)	0.0013 (10)	0.0004 (10)	−0.0006 (10)
C12	0.0248 (13)	0.0190 (13)	0.0215 (13)	0.0009 (11)	0.0043 (11)	−0.0014 (10)
C13	0.0216 (13)	0.0221 (14)	0.0118 (11)	−0.0003 (10)	−0.0067 (10)	−0.0014 (10)
C14	0.0097 (10)	0.0178 (12)	0.0098 (11)	−0.0005 (9)	−0.0020 (9)	−0.0006 (9)
C15	0.0176 (12)	0.0172 (13)	0.0123 (11)	0.0003 (10)	−0.0009 (10)	−0.0010 (9)
C16	0.0195 (12)	0.0208 (13)	0.0135 (12)	−0.0031 (10)	0.0006 (10)	0.0042 (10)
C17	0.0138 (11)	0.0278 (14)	0.0117 (11)	0.0012 (10)	0.0011 (10)	−0.0001 (10)
C18	0.0113 (11)	0.0189 (12)	0.0157 (12)	0.0032 (9)	−0.0018 (9)	−0.0022 (10)
C19	0.0079 (10)	0.0164 (12)	0.0143 (11)	0.0015 (9)	−0.0033 (9)	0.0019 (9)
C20	0.0168 (12)	0.0136 (12)	0.0099 (11)	−0.0014 (9)	−0.0021 (9)	0.0008 (9)
C21	0.0161 (12)	0.0167 (13)	0.0135 (11)	0.0002 (9)	0.0003 (9)	0.0019 (9)
C22	0.0292 (14)	0.0149 (12)	0.0127 (12)	0.0032 (10)	0.0027 (11)	0.0005 (9)
C23	0.0310 (14)	0.0143 (12)	0.0130 (12)	−0.0059 (11)	−0.0059 (11)	0.0013 (10)
C24	0.0180 (13)	0.0258 (14)	0.0147 (12)	−0.0075 (10)	−0.0048 (10)	0.0032 (10)
C25	0.0160 (12)	0.0211 (13)	0.0129 (11)	0.0001 (10)	−0.0003 (9)	0.0002 (10)

### Geometric parameters (Å, º)

**Table:**

Pd1—C13	2.048 (2)	C11—C12	1.358 (4)
Pd1—N1	2.147 (2)	C11—H11	0.9500
Pd1—P1	2.1965 (6)	C12—H12	0.9500
Pd1—Cl1	2.3761 (6)	C13—H13A	0.9800
S1—C12	1.717 (3)	C13—H13B	0.9800
S1—C9	1.736 (2)	C13—H13C	0.9800
P1—C20	1.817 (2)	C14—C19	1.394 (3)
P1—C14	1.822 (2)	C14—C15	1.399 (3)
P1—C1	1.832 (2)	C15—C16	1.389 (3)
N1—C7	1.273 (3)	C15—H15	0.9500
N1—C8	1.490 (3)	C16—C17	1.386 (4)
C1—C6	1.393 (4)	C16—H16	0.9500
C1—C2	1.416 (3)	C17—C18	1.388 (4)
C2—C3	1.404 (3)	C17—H17	0.9500
C2—C7	1.467 (3)	C18—C19	1.390 (3)
C3—C4	1.377 (4)	C18—H18	0.9500
C3—H3	0.9500	C19—H19	0.9500
C4—C5	1.386 (4)	C20—C21	1.398 (3)
C4—H4	0.9500	C20—C25	1.399 (3)
C5—C6	1.390 (3)	C21—C22	1.382 (4)
C5—H5	0.9500	C21—H21	0.9500
C6—H6	0.9500	C22—C23	1.388 (4)
C7—H7	0.9500	C22—H22	0.9500
C8—C9	1.498 (3)	C23—C24	1.381 (4)
C8—H8A	0.9900	C23—H23	0.9500
C8—H8B	0.9900	C24—C25	1.389 (4)
C9—C10	1.375 (4)	C24—H24	0.9500
C10—C11	1.428 (4)	C25—H25	0.9500
C10—H10	0.9500		
			
C13—Pd1—N1	178.18 (9)	C12—C11—C10	113.7 (2)
C13—Pd1—P1	89.57 (8)	C12—C11—H11	123.2
N1—Pd1—P1	90.23 (6)	C10—C11—H11	123.2
C13—Pd1—Cl1	86.45 (8)	C11—C12—S1	111.2 (2)
N1—Pd1—Cl1	93.96 (6)	C11—C12—H12	124.4
P1—Pd1—Cl1	171.99 (2)	S1—C12—H12	124.4
C12—S1—C9	92.29 (12)	Pd1—C13—H13A	109.5
C20—P1—C14	105.43 (11)	Pd1—C13—H13B	109.5
C20—P1—C1	105.36 (11)	H13A—C13—H13B	109.5
C14—P1—C1	100.70 (10)	Pd1—C13—H13C	109.5
C20—P1—Pd1	115.88 (8)	H13A—C13—H13C	109.5
C14—P1—Pd1	117.03 (8)	H13B—C13—H13C	109.5
C1—P1—Pd1	110.82 (8)	C19—C14—C15	119.8 (2)
C7—N1—C8	116.0 (2)	C19—C14—P1	119.31 (18)
C7—N1—Pd1	128.02 (17)	C15—C14—P1	120.75 (18)
C8—N1—Pd1	115.98 (15)	C16—C15—C14	119.9 (2)
C6—C1—C2	118.9 (2)	C16—C15—H15	120.1
C6—C1—P1	119.66 (18)	C14—C15—H15	120.1
C2—C1—P1	121.07 (18)	C17—C16—C15	120.2 (2)
C3—C2—C1	118.5 (2)	C17—C16—H16	119.9
C3—C2—C7	114.6 (2)	C15—C16—H16	119.9
C1—C2—C7	126.7 (2)	C16—C17—C18	120.1 (2)
C4—C3—C2	121.6 (2)	C16—C17—H17	120.0
C4—C3—H3	119.2	C18—C17—H17	120.0
C2—C3—H3	119.2	C17—C18—C19	120.2 (2)
C3—C4—C5	119.9 (2)	C17—C18—H18	119.9
C3—C4—H4	120.0	C19—C18—H18	119.9
C5—C4—H4	120.0	C18—C19—C14	119.9 (2)
C4—C5—C6	119.5 (2)	C18—C19—H19	120.1
C4—C5—H5	120.2	C14—C19—H19	120.1
C6—C5—H5	120.2	C21—C20—C25	119.3 (2)
C5—C6—C1	121.5 (2)	C21—C20—P1	119.21 (18)
C5—C6—H6	119.2	C25—C20—P1	120.93 (18)
C1—C6—H6	119.2	C22—C21—C20	120.2 (2)
N1—C7—C2	128.7 (2)	C22—C21—H21	119.9
N1—C7—H7	115.6	C20—C21—H21	119.9
C2—C7—H7	115.6	C21—C22—C23	120.3 (2)
N1—C8—C9	110.1 (2)	C21—C22—H22	119.9
N1—C8—H8A	109.6	C23—C22—H22	119.9
C9—C8—H8A	109.6	C24—C23—C22	119.9 (2)
N1—C8—H8B	109.6	C24—C23—H23	120.0
C9—C8—H8B	109.6	C22—C23—H23	120.0
H8A—C8—H8B	108.2	C23—C24—C25	120.4 (2)
C10—C9—C8	128.0 (2)	C23—C24—H24	119.8
C10—C9—S1	110.83 (19)	C25—C24—H24	119.8
C8—C9—S1	121.11 (18)	C24—C25—C20	119.9 (2)
C9—C10—C11	112.0 (2)	C24—C25—H25	120.1
C9—C10—H10	124.0	C20—C25—H25	120.1
C11—C10—H10	124.0		
			
C13—Pd1—P1—C20	−25.48 (12)	N1—C8—C9—C10	88.4 (3)
N1—Pd1—P1—C20	156.33 (10)	N1—C8—C9—S1	−87.7 (2)
Cl1—Pd1—P1—C20	34.72 (19)	C12—S1—C9—C10	0.2 (2)
C13—Pd1—P1—C14	99.93 (11)	C12—S1—C9—C8	176.9 (2)
N1—Pd1—P1—C14	−78.26 (10)	C8—C9—C10—C11	−176.7 (2)
Cl1—Pd1—P1—C14	160.13 (16)	S1—C9—C10—C11	−0.3 (3)
C13—Pd1—P1—C1	−145.43 (11)	C9—C10—C11—C12	0.3 (3)
N1—Pd1—P1—C1	36.37 (10)	C10—C11—C12—S1	−0.1 (3)
Cl1—Pd1—P1—C1	−85.23 (18)	C9—S1—C12—C11	−0.1 (2)
C13—Pd1—N1—C7	−113 (3)	C20—P1—C14—C19	147.04 (18)
P1—Pd1—N1—C7	−29.4 (2)	C1—P1—C14—C19	−103.59 (19)
Cl1—Pd1—N1—C7	143.8 (2)	Pd1—P1—C14—C19	16.6 (2)
C13—Pd1—N1—C8	66 (3)	C20—P1—C14—C15	−37.7 (2)
P1—Pd1—N1—C8	149.87 (16)	C1—P1—C14—C15	71.7 (2)
Cl1—Pd1—N1—C8	−36.96 (16)	Pd1—P1—C14—C15	−168.13 (16)
C20—P1—C1—C6	28.9 (2)	C19—C14—C15—C16	−0.8 (3)
C14—P1—C1—C6	−80.5 (2)	P1—C14—C15—C16	−176.12 (18)
Pd1—P1—C1—C6	154.97 (17)	C14—C15—C16—C17	1.6 (4)
C20—P1—C1—C2	−158.43 (18)	C15—C16—C17—C18	−0.9 (4)
C14—P1—C1—C2	92.1 (2)	C16—C17—C18—C19	−0.6 (4)
Pd1—P1—C1—C2	−32.4 (2)	C17—C18—C19—C14	1.4 (3)
C6—C1—C2—C3	−0.2 (3)	C15—C14—C19—C18	−0.7 (3)
P1—C1—C2—C3	−172.87 (17)	P1—C14—C19—C18	174.69 (17)
C6—C1—C2—C7	174.5 (2)	C14—P1—C20—C21	155.04 (18)
P1—C1—C2—C7	1.8 (3)	C1—P1—C20—C21	49.0 (2)
C1—C2—C3—C4	−0.6 (4)	Pd1—P1—C20—C21	−73.8 (2)
C7—C2—C3—C4	−175.9 (2)	C14—P1—C20—C25	−33.8 (2)
C2—C3—C4—C5	0.7 (4)	C1—P1—C20—C25	−139.78 (19)
C3—C4—C5—C6	−0.1 (4)	Pd1—P1—C20—C25	97.34 (19)
C4—C5—C6—C1	−0.7 (4)	C25—C20—C21—C22	2.0 (4)
C2—C1—C6—C5	0.8 (4)	P1—C20—C21—C22	173.29 (18)
P1—C1—C6—C5	173.59 (19)	C20—C21—C22—C23	−0.7 (4)
C8—N1—C7—C2	−174.8 (2)	C21—C22—C23—C24	−1.0 (4)
Pd1—N1—C7—C2	4.4 (4)	C22—C23—C24—C25	1.4 (4)
C3—C2—C7—N1	−168.7 (2)	C23—C24—C25—C20	−0.1 (4)
C1—C2—C7—N1	16.5 (4)	C21—C20—C25—C24	−1.6 (4)
C7—N1—C8—C9	104.4 (2)	P1—C20—C25—C24	−172.73 (19)
Pd1—N1—C8—C9	−74.9 (2)		
